# Epigenotype, Genotype, and Phenotype Analysis of Taiwanese Patients with Silver–Russell Syndrome

**DOI:** 10.3390/jpm11111197

**Published:** 2021-11-13

**Authors:** Hsiang-Yu Lin, Chung-Lin Lee, Sisca Fran, Ru-Yi Tu, Ya-Hui Chang, Dau-Ming Niu, Chia-Ying Chang, Pao-Chin Chiu, Yen-Yin Chou, Hui-Pin Hsiao, Meng-Che Tsai, Mei-Chyn Chao, Li-Ping Tsai, Chia-Feng Yang, Pen-Hua Su, Yu-Wen Pan, Chen-Hao Lee, Tzu-Hung Chu, Chih-Kuang Chuang, Shuan-Pei Lin

**Affiliations:** 1 Department of Medicine, MacKay Medical College, New Taipei City 25245, Taiwan; lxc46199@ms37.hinet.net (H.-Y.L.); clampcage@yahoo.com.tw (C.-L.L.); 2 Department of Pediatrics, MacKay Memorial Hospital, Taipei 10449, Taiwan; wish1001026@gmail.com; 3 Department of Medical Research, MacKay Memorial Hospital, Taipei 10449, Taiwan; fransiscazen@gmail.com (S.F.); likemaruko@hotmail.com (R.-Y.T.); 4 MacKay Junior College of Medicine, Nursing and Management, Taipei 10449, Taiwan; 5 Department of Medical Research, China Medical University Hospital, China Medical University, Taichung 40402, Taiwan; 6 Department of Rare Disease Center, MacKay Memorial Hospital, Taipei 10449, Taiwan; 7 Institute of Clinical Medicine, National Yang-Ming University, Taipei 11221, Taiwan; dmniu1111@yahoo.com.tw; 8 Institute of Clinical Medicine, National Yang-Ming Chiao Tung University, Taipei 11221, Taiwan; 9 Department of Pediatrics, Taipei Veterans General Hospital, Taipei 11217, Taiwan; pum_chia@yahoo.com.tw; 10 Department of Pediatrics, MacKay Memorial Hospital, Hsinchu 300, Taiwan; ailxjdau@yahoo.com.tw; 11 Department of Pediatrics, Kaohsiung Veterans General Hospital, Kaohsiung 800, Taiwan; paochinped333@gmail.com; 12 Department of Pediatrics, National Cheng Kung University Hospital, College of Medicine, National Cheng Kung University, Tainan 70101, Taiwan; yenyin@mail.ncku.edu.tw (Y.-Y.C.); ache93@yahoo.com.tw (M.-C.T.); panyuwen0527@hotmail.com (Y.-W.P.); 13 Department of Pediatrics, Kaohsiung Medical University Chung Ho Memorial Hospital, Kaohsiung 807, Taiwan; hsiaohb@yahoo.com.tw; 14 Department of Pediatrics, Changhua Christian Children’s Hospital, Changhua 500, Taiwan; mcchao1@gmail.com; 15 Department of Pediatrics, Taipei Tzu Chi Hospital, Buddhist Tzu Chi Medical Foundation, New Taipei City 231, Taiwan; tsaiped@gmail.com; 16 Department of Medicine, Tzu Chi University, Hualian 970, Taiwan; 17 Department of Pediatrics, Chung Shan Medical University, Taichung 40201, Taiwan; ninaphsu@gmail.com; 18 Department of Pediatrics, E-DA Hospital, I-Shou University, Kaohsiung 824, Taiwan; jacklee1005@hotmail.com; 19 Children Growth & Development Department, IHMED International Medical, Taipei 108, Taiwan; mno4.chu@gmail.com; 20 College of Medicine, Fu-Jen Catholic University, Taipei 24205, Taiwan; 21 Department of Infant and Child Care, National Taipei University of Nursing and Health Sciences, Taipei 11219, Taiwan

**Keywords:** Silver–Russell syndrome, epigenotype, genotype, methylation, phenotype

## Abstract

Background: Silver–Russell syndrome (SRS) is a clinically and genetically heterogeneous disorder characterized by severe intrauterine growth retardation, poor postnatal growth, characteristic facial features, and body asymmetry. Hypomethylation of the imprinted genes of the chromosome 11p15.5 imprinting gene cluster and maternal uniparental disomy of chromosome 7 (mUPD7) are the major epigenetic disturbances. The aim of this study was to characterize the epigenotype, genotype, and phenotype of these patients in Taiwan. Methods: Two hundred and six subjects with clinically suspected SRS were referred for diagnostic testing, which was performed by profiling the methylation of *H19*-associated imprinting center (IC) 1 and the imprinted *PEG1/MEST* region using methylation-specific multiplex ligation-dependent probe amplification and high-resolution melting analysis with a methylation-specific polymerase chain reaction assay. We also applied a whole genome strategy to detect copy number changes and loss of heterozygosity. Clinical manifestations were recorded and analyzed according to the SRS scoring system proposed by Bartholdi et al. Results: Among the 206 referred subjects, 100 were classified as having a clinical diagnosis of SRS (score ≥ 8, maximum = 15) and 106 had an SRS score ≤ 7. Molecular lesions were detected in 45% (45/100) of the subjects with a clinical diagnosis of SRS, compared to 5% (5/106) of those with an SRS score ≤ 7. Thirty-seven subjects had IC1 hypomethylation, ten subjects had mUPD7, and three subjects had microdeletions. Several clinical features were found to be statistically different (*p* < 0.05) between the “IC1 hypomethylation” and “mUPD7” groups, including relative macrocephaly at birth (89% vs. 50%), triangular shaped face (89% vs. 50%), clinodactyly of the fifth finger (68% vs. 20%), and SRS score (11.4 ± 2.2 vs. 8.3 ± 2.5). Conclusions: The SRS score was positively correlated with the molecular diagnosis rate (*p* < 0.001). The SRS subjects with mUPD7 seemed to have fewer typical features and lower SRS scores than those with IC1 hypomethylation. Careful clinical observation and timely molecular confirmation are important to allow for an early diagnosis and multidisciplinary management of these patients.

## 1. Introduction

Silver–Russellsyndrome (SRS; OMIM #180860) is a clinically and genetically heterogeneous disorder characterized by severe intrauterine growth retardation, poor postnatal growth, relative macrocephaly, a triangle-shaped face with a prominent forehead and small chin, body and limb asymmetry, and fifth finger clinodactyly. It was first described independently by Silver et al. [1] and Russell et al. [2] in 1953 and 1954, respectively. Silver et al. [1] described two cases with hemihypertrophy and short stature, and Russell et al. [2] reported five similar children with intrauterine dwarfism recognizable at birth, craniofacial dysostosis, and disproportionately short arms. However, the relatively non-specific features of SRS make the clinical diagnosis challenging. The prevalence of SRS has been estimated to range from 1:30,000 to 1:100,000, however it may be underestimated because of the variable and diverse clinical presentations. Most SRS cases are sporadic, although occasional familial cases have been reported [3,4].

Hypomethylation of imprinting center 1 (IC1) on the paternal allele of the chromosome 11p15 region that regulates the *IGF2/H19* locus and maternal uniparental disomy of chromosome 7 (mUPD7) are the two major epigenetic disturbances, found in about 30–60% and 5–10% of SRS cases, respectively. Rare cytogenetic rearrangements have also been reported in 1% to 2% of cases. Hypermethylation of IC2, copy number variations, and sequence variants in *IGF2, CDKN1C, HMGA2*, or *PLAG1* were recently identified as (epi)genetic alterations associated with SRS [3,4,5]. However, a substantial proportion of SRS patients have an unknown genetic etiology [6,7,8,9,10].

In clinical practice, pre- and postnatal growth retardation are relatively common and non-specific conditions caused by several genetic backgrounds, as well as maternal or other environmental factors. Therefore, comprehensive phenotypic records before genetic testing for SRS are important. Molecular analysis can help to classify individuals with SRS into subgroups, which can provide additional information on the natural course and prognosis as well as genetic counseling for the patients and their families.

Phenotype and genotype/epigenotype correlations in European and North American SRS patients have been reported in the literature [6,8,9,11,12,13]. For example, SRS patients with mUPD7 have been shown to be phenotypically milder than those with IC1 hypomethylation and other idiopathic SRS cases, with pre- and postnatal growth restriction and mild or absent SRS craniofacial dysmorphology [14]. However, there are only limitedreports of the molecular basis and clinical features of SRS inAsia [4,5,15]. Therefore, the aim of this study was to characterize the epigenotype, genotype, and phenotype of Taiwanese patients with SRS.

## 2. Patients and Methods

### 2.1. Study Population

We performed a retrospective chart review of 206 subjects with clinically suspected SRS (98 males and 108 females; age range, 1 day to 23 years) who were referred for diagnostic testing from January 2007 through June 2021 at MacKay Memorial Hospital, Taipei, Taiwan. All information was obtained from the medical records. The chart review was performed by a single author (HYL) to ensure consistent extraction of information. Written informed consent was obtained from a parent if the patient was a child and from the patient if they were over 18 years of age. The study was approved by the Ethics Committee of MacKay Memorial Hospital, Taipei, Taiwan.

### 2.2. Clinical Assessments

**Figure 1:**
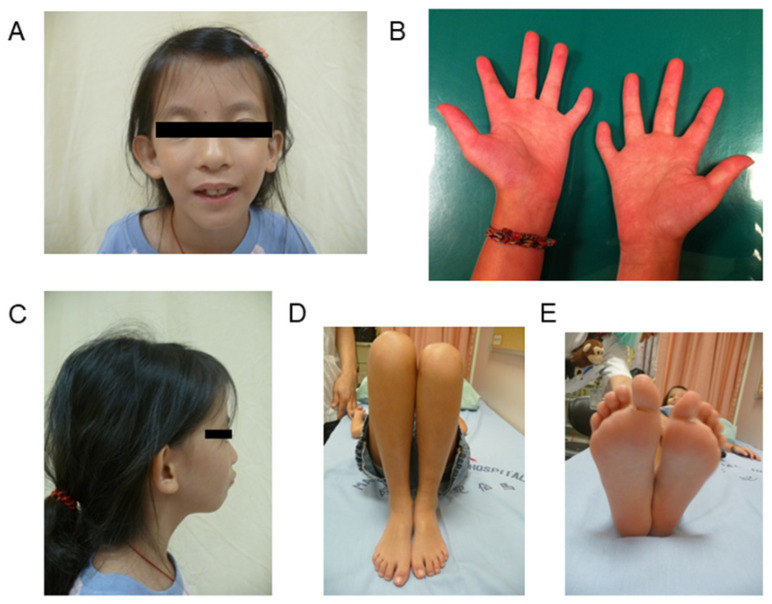
Clinical features of a 5-year-old girl with Silver–Russell syndrome (SRS). She had IC1 hypomethylation and an SRS score of 12. (**A**) Triangular shaped face. (**B**) Clinodactyly of bilateral fifth fingers and right hand larger than the left hand. (**C**) Small chin. (**D**) Right leg longer than the left leg. (**E**) Right foot larger than the left foot. IC, imprinting center.

Clinical manifestations were recorded and analyzed according to the diagnostic criteria proposed by Bartholdi et al. [11]. Each patient was scored for the following five domains: (1) parameters at birth; (2) postnatal course; (3) asymmetry; (4) facial features; (5) other features. Asymmetry was evaluated simply as being present (3 points) or absent (0 points). For the other four domains, three features were scored from 0–3 points, with 0 indicating that none of the three features was present, 1 indicating that one feature was present, etc. According to this ‘‘SRS severity’’ scoring system, the minimum total score was 0 points and the maximum total score was 15 points. Subjects with a score ≥ 8 points were classified as having SRS [11] (Figure 1). Other data acquired from the records included sex, age at diagnosis, height and weight standard deviation scores (*z* scores) at diagnosis, history of conception by assisted reproductive technology (ART), gestational age at birth, and parental age at childbirth, as well as birth length, weight, and head circumference *z* scores. *Z* scores for height and weight were calculated using standard growth tables for the Taiwanese population [16]. A *z* score was derived by subtracting the population mean from each individual’s raw score, and then dividing the difference by the standard deviation of the population.

### 2.3. Molecular Studies

All DNA was extracted from peripheral blood using a Chemagic DNA Blood Kit (Chemagen, Baesweiler, Germany) and a MethylCode Bisulfite Conversion Kit (Invitrogen, Carlsbad, CA, USA) according to the manufacturer’s instructions. All diagnostic examinations were performedby profiling the methylation of *H19*-associated IC1 using methylation-specific multiplex ligation-dependent probe amplification (MS-MLPA) and methylation-specific high-resolution melting (MS-HRM) analysis with a methylation-specific polymerase chain reaction assay. mUPD7 was tested by analyzing the methylation status of the imprinted *PEG1/MEST* region using MS-MLPA and MS-HRM analysis. In addition, we used a whole genome strategy to detect copy number changes and loss of heterozygosity. The detailed procedures have been described previously [17,18,19].

### 2.4. Data and Statistical Analysis

For the subjects with a clinical diagnosis of SRS (score ≥ 8), we compared the clinical features and SRS scores between subjects with identified molecular defects versus those without identified molecular defects, as well as those with IC1 hypomethylation versus those with mUPD7 using Student’s *t*-test for continuous variables and Fisher’s exact test for categorical variables. Two-tailed *p*-values were computed. The relationship between molecular diagnosis rate and SRS score of the 206 subjects was evaluated using Pearson’s correlation coefficient (*r*), and testing for statistical significance (*p* < 0.05) was performed using Fisher’s *r-z* transformations. All statistical analyses were conducted using SPSS version 11.5 (SPSS Inc., Chicago, IL, USA). Statistical significance was set at *p* < 0.05.

## 3. Results

**Figure 2:**
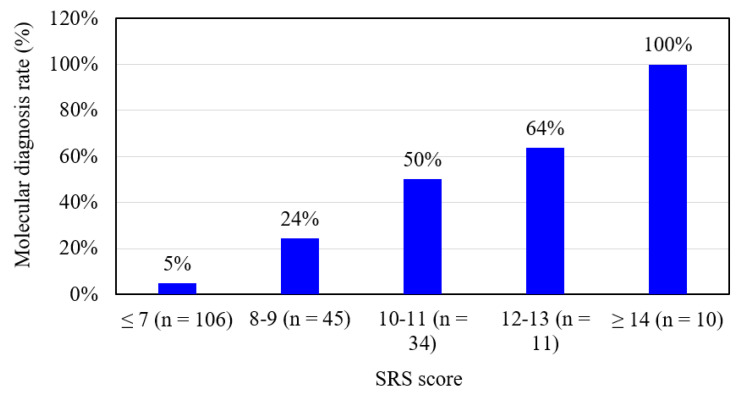
The molecular diagnosis rates of the 206 subjects with a clinical diagnosis of Silver–Russell syndrome (SRS) (SRS score ≥ 8) (*n* = 100) or an SRS score ≤ 7 (*n* = 106) divided into five groups according to SRS score (maximum = 15).

Among the 206 referred subjects, 100 were classified as having a clinical diagnosis of SRS (score ≥ 8, maximum = 15) and 106 had an SRS score ≤ 7. Molecular lesions were detected in 45% (45/100) of the subjects with a clinical diagnosis of SRS (score ≥ 8), compared to 5% (5/106) of those with an SRS score ≤ 7. Thirty-seven subjects had IC1 hypomethylation and 10 subjects had mUPD7. The whole genome arrays identified that three subjects had microdeletions on chromosomes 22q11.21q11.22 (1.497 Mb deletion), 6q24.2q25.2 (10.47 Mb deletion), and 13q31.3 (1.73 Mb deletion) with SRS scores of 7, 8, and 10, respectively (Table 1). Table 2 shows that the subjects with IC1 hypomethylation (*n* = 37, median score = 11) had a more severe phenotype than those with mUPD7 (*n* = 10, median score = 8), microdeletions (*n* = 3, median score = 8), and the idiopathic SRS patients (*n* = 55, median score = 9). The molecular diagnosis rate was positively correlated with the SRS score (*r* = 0.572, *p* < 0.001). To investigate this trend, we further divided the 206 subjects into five groups according to the SRS score. The molecular diagnosis rates were 5% (≤7 points, *n* = 106), 24% (8–9 points, *n* = 45), 50% (10–11 points, *n* = 34), 64% (12–13 points, *n* = 11), and 100% (≥14 points, *n* = 10) (Figure 2).

Among the 100 subjects with a clinical diagnosis of SRS (score ≥ 8), birth weight ≤ the 10th centile was the most common manifestation (91%), followed by birth length ≤ the 10th centile (90%), and postnatal height ≤ the 3rd centile (90%). Several clinical features were found to be statistically different (*p* < 0.05) between the “with identified molecular defect (*n* = 43)” and “without identified molecular defect (*n* = 57)” groups, including height *z* score at diagnosis (−3.1 ± 1.6 vs. −2.6 ± 1.2), birth length *z* score (−4.1 ± 1.6 vs. −2.5 ± 1.4), birth weight *z* score (−3.0 ± 0.9 vs. −2.4 ± 1.0), birth weight ≤ the 10th centile (98% vs. 86%), birth length ≤ the 10th centile (98% vs. 84%), relative macrocephaly at birth (86% vs. 33%), postnatal normal head circumference (88% vs. 60%), normal cognitive development (86% vs. 68%), asymmetry (77% vs. 56%), and total SRS score (11.3 ± 2.1 vs. 9.4 ± 1.2; maximum = 15) (Table 3). Table 3 also shows the clinical characteristics of 39 SRS patients reported by Netchine et al. [8].

Table 4 shows the clinical features of 47 subjects identified to have molecular defects, including 37 subjects with IC1 hypomethylation and 10 with mUPD7. The mean age at diagnosis was 4.4 years for those with IC1 hypomethylation, and 6.1 years for those with mUPD7. Comparing the birth data between the IC1 hypomethylation and mUPD7 groups, the IC1 hypomethylation group had lower mean birth length and weight, but higher mean head circumference than the mUPD7 group. Several clinical features were found to be statistically different (*p* < 0.05) between the IC1 hypomethylation and mUPD7 groups, including gestational age (38.0 ± 1.7 weeks vs. 36.6 ± 1.6 weeks), paternal age at childbirth (32.9 ± 4.3 vs. 37.6 ± 4.8), maternal age at childbirth (30.3 ± 4.1 vs. 35.3 ± 5.1), relative macrocephaly at birth (89% vs. 50%), triangular shaped face (89% vs. 50%), clinodactyly of the fifth finger (68% vs. 20%), and total SRS score (11.4 ± 2.2 vs. 8.7 ± 2.5; maximum = 15).

**Figure 3:**
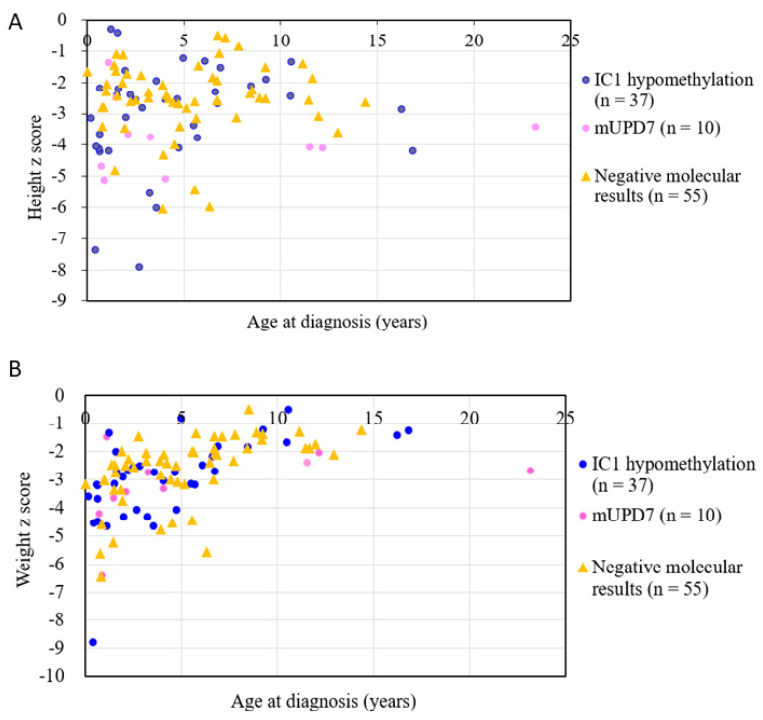
The relationships between the age at diagnosis and (**A**) height *z* score (*n* = 102, *r* = 0.069, *p* > 0.05) and (**B**) weight *z* score (*n* = 102, *r* = 0.509, *p* < 0.001) among 47 subjects with identified molecular defects (37 subjects with IC1 hypomethylation and 10 subjects with mUPD7) and 55 subjects with an SRS score ≥ 8 and negative molecular results. SRS: Silver–Russell syndrome; IC, imprinting center; mUPD7: maternal uniparental disomy of chromosome 7.

Figure 3 shows the relationships between the age at diagnosis and height *z* score and weight *z* score among the 47 subjects with identified molecular defects (37 subjects with IC1 hypomethylation and 10 subjects with mUPD7) and 55 subjects with SRS score ≥ 8 and negative molecular results. The age at diagnosis was positively correlated with the weight *z* score (*r* = 0.509, *p* < 0.001).

Among the 100 subjects with a clinical diagnosis of SRS (score ≥ 8), five subjects (5%) were conceived byART. Three of these subjects were identified to have IC1 hypomethylation with SRS scores of 8, 9, and 11. The other two subjects had normal molecular study results and their SRS scores were both 11.

## 4. Discussion

A number of studies have described the clinical and molecular findings for Western European patients with SRS [6,8,9,10,11,12,13,14]; however, only a few studies have been conducted in Asian patients [4,5,15]. To the best of our knowledge, this is the first comprehensive clinical and epigenetic study to analyze the epigenotype, genotype, and phenotype of Taiwanese patients with SRS. SRS is primarily a clinical diagnosis; however, molecular analysis helps to confirm the clinical diagnosis and classify the subtype [3]. In our cohort of 100 subjects with a clinical diagnosis of SRS (score ≥ 8), the overall molecular defect detection rate was 45%. IC1 hypomethylation, mUPD7, and microdeletion accounted for 36%, 7%, and 2% of the cases, respectively. Our results are consistent with those reported previously [8,9,15].

Bartholdi et al. [11] reported that patients with IC1 hypomethylation (*n* = 11, median score = 11) had a more severe phenotype than those with mUPD7 (*n* = 10, median score = 8) and idiopathic SRS patients (*n* = 58, median score = 9) according to their SRS severity scoring system, indicating that IC1 hypomethylation was associated with a severe SRS phenotype. Our results are consistent with theirs.

Fuke et al. [15] analyzed the epigenotype/phenotype in their cohort, and revealed that patients with IC1 hypomethylation had a greater reduction in birth length and weight, more preserved birth occipitofrontal circumference, and more frequent body asymmetry than patients with mUPD7. In addition, Wakeling et al. [6] reported that asymmetry, fifth finger clinodactyly and congenital anomalies were more commonly seen in subjects with IC1 hypomethylation, whereas learning difficulties and referral for speech therapy were more common in subjects with mUPD7. In our cohort, the frequencies of these items between the IC1 hypomethylation and mUPD7 groups were similar to those of their studies. Bartholdi et al. [11] reported that body asymmetry was more common in SRS patients with IC1 hypomethylation (65%) compared to idiopathic SRS patients (40%), those with mUPD7 (20%), and those without SRS (5%). They found that mUPD7 was less likely to result in typical SRS features. Our results are consistent with those of the previous reports [6,8,9,10,11,15].

Wakeling et al. [6] reported that patients with IC1 hypomethylation were less likely to show a postnatal reduction in height *z* scores than those with mUPD7, although the number of cases was too small to reach statistical significance. We also found similar findings in our patients.

Lin et al. [17] reported that the whole genome approach could provide information on the etiology of SRS, and that if no epimutations are identified in patients with typical SRS, microdeletions should be suspected. We identified three patients with microdeletions in our cohort and they had SRS scores of 7, 8, and 10. Since many microdeletion syndromes present with growth retardation and dysmorphism, which overlap with SRS, health care professionals should keep in mind the possibility of microdeletions when no epimutations are detected in patients with SRS [17].

Netchine et al. [8] reported that birth weight and length were significantly lower, and a prominent forehead, body asymmetry, and relative macrocephaly were significantly more frequent in SRS patients with IC1 hypomethylation than in those without IC1 hypomethylation. Our results are consistent with theirs. Netchine et al. also reported individual clinical features in 39 SRS patients, of which postnatal height ≤ the 3rd centile (92.3%), bossing forehead (92.3%), and relative macrocephaly at birth (84.6%) were the most common. In our 100 patients with clinically diagnosed SRS, birth weight ≤ the 10th centile was the most common manifestation (91%), followed by birth length ≤ the 10th centile (90%), and postnatal height ≤ the 3rd centile (90%).

In the present study, the molecular diagnosis rate was positively correlated with the SRS score (*p* < 0.01). Our results demonstrate the feasibility of using the SRS severity scoring system developed by Bartholdi et al. [11] to predict outcomes of molecular abnormalities.

For the 100 subjects with a clinical diagnosis of SRS (score ≥ 8) in the present study, we delineated the relationships between the age at diagnosis and height *z* score and weight *z* score, respectively. We found that the age at diagnosis was positively correlated with weight *z* score (*p* < 0.001). The age at diagnosis also had a positive trend with height *z* score, although the *p* values did not reach statistical significance. There are two possible explanations for these findings. First, the patients with an earlier age at diagnosis were usually more typical SRS cases, meaning lower height *z* score and lower weight *z* score than those with an older age at diagnosis. Second, the characteristic SRS features seem to change and are not as significant in late childhood and adulthood [8].

Advanced maternal age at childbirth has been shown to be a predisposing factor for the development of mUPD15 because of increased non-disjunction at meiosis 1 [20]. However, there are few such studies for mUPD7, primarily due to the relative rarity of mUPD7. In our cohort, maternal age at childbirth in the mUPD7 patients was significantly higher than that in the IC1 hypomethylation patients. In addition, we also found a higher paternal age in the mUPD7 group.

ART has been reported to be associated with higher incidence rates of normally rare imprinting disorders such as SRS, Beckwith–Wiedemann syndrome, Prader–Willi syndrome, and Angelman syndrome [21]. A nationwide epidemiological study from 1985 through 2015 in Japan conducted by Hattori et al. [22] revealed an 8.91-fold increased frequency of SRS associated with ART, and 11.9% (8/67) of their subjects were found to have SRS. They concluded that the imprinting disorders related to ART may tend to occur just after fertilization, at a time when the epigenome is most vulnerable and may be affected by the techniques of manipulation used for in vitro fertilization or intracytoplasmic sperm injection and the culture medium of the fertilized egg. In addition, Wakeling et al. [6] reported that the use of ART seemed to increase IC1 hypomethylation compared with the general population. In our cohort, five SRS subjects (5/100, 5%) were conceived byART, and IC1 hypomethylation was detected in three of them. Our results are in agreement with the previous reports.

MS-HRM has been reported to be a rapid, cost-effective, and sensitive method for screening methylation changes at the *H19* loci in human imprinting disorders, including SRS [19]. MS-MLPA analysis is recommended as the first-line test for the molecular diagnosis of SRS [18]. In the present study, we used the molecular methods that were qualified for the molecular diagnosis of SRS.

### Limitations

As a retrospective and cross-sectional study, not all clinical data were available for all of our subjects. Due to the limitation of the study design in 2012, the Bartholdi clinical scoring system (in 2009) [11] was used instead of the Netchine–Harbison clinical scoring system (in 2017) [3] in this study. The relatively small sample size of those with molecular-confirmed SRS with different etiologies reflects the rare nature of this genetic disorder. Due to the limitation of the study design, none of the monogenic causes of SRS have been analyzed in this cohort. In addition, the degree of disease severity was quite wide, as was the age range. Thus, further studies with larger cohorts and a longer follow-up period are warranted.

## 5. Conclusions

This 14-year review is the first comprehensive clinical and epigenetic study of SRS patients in Taiwan. The results show that the clinical features and underlying epigenetic mechanisms of Taiwanese SRS patients are similar to those of other Western populations. The SRS score was positively correlated with the molecular diagnosis rate. The SRS subjects with mUPD7 seemed to have fewer typical features and lower SRS scores than those with IC1 hypomethylation. Careful clinical observation and timely molecular confirmation are important to allow for an early diagnosis and multidisciplinary management of these patients.

## Figures and Tables

**Table 1:** Epigenetic and genetic defects of 206 subjects with a clinical diagnosis of SRS (score ≥ 8, *n* = 100) or SRS score ≤ 7 (*n* = 106).

Clinical Classification	SRS Score (Maximum = 15)	Epigenetic and Genetic Defects				
		IC1 Hypomethylation (%)	mUPD7 (%)	Microdeletion	Unknown (%)	Molecular Diagnosis Rate
Clinical diagnosis with SRS (score≥ 8) (*n* = 100)	10.2 ± 1.9	36 (36%)	7 (7%)	2 (2%)	55 (55%)	45%
Subjects with SRS score ≤ 7 (*n* = 106)	5.3 ± 1.5	1 (1%)	3 (3%)	1 (1%)	101 (95%)	5%

SRS, Silver–Russell syndrome; IC, imprinting center; mUPD7, maternal uniparental disomy of chromosome 7.

**Table 2:** Epigenetic and genetic defects and molecular diagnosis rates of the 206 subjects by SRS score.

SRS Score (Maximum = 15)	*n*	IC1 Hypomethylation	mUPD7	Microdeletion	Unknown	Molecular Diagnosis	Molecular Diagnosis Rate (%)
≤ 7	106	1	3	1	101	5	5%
8	21	2	3	1	15	6	29%
9	24	5	0	0	19	5	21%
10	17	2	3	1	11	6	35%
11	17	11	0	0	6	11	65%
12	8	5	0	0	3	5	63%
13	3	2	0	0	1	2	67%
14	8	7	1	0	0	8	100%
15	2	2	0	0	0	2	100%
All	206	37	10	3	156	50	24%

SRS, Silver–Russell syndrome; IC, imprinting center; mUPD7, maternal uniparental disomy of chromosome 7.

**Table 3:** Clinical features of 100 subjects with a clinical diagnosis of SRS with and without molecular defects in the present study compared with those in the report of Netchine et al. [8] (*n* = 39).

Clinical Features	Clinical Diagnosis (*n* = 100)		*p* Value	Clinical Diagnosis (*n* = 100)	Netchine et al. [8] (*n* = 39)
	With Identified Molecular Defect (*n* = 43)	Without Identified Molecular Defect (*n* = 57)			
Male/Female	25/18	26/31	0.219	51/49	19/20
Age at diagnosis (years)	4.8 ± 5.0	5.2 ± 3.5	0.717	5.0 ± 4.2	—
Height *z* score at diagnosis	−3.1 ± 1.6	−2.6 ± 1.2	**0.045**	−2.8 ± 1.4	−3.6 ± 1.5
Weight *z* score at diagnosis	−3.0 ± 1.4	−2.7 ± 1.2	0.262	−2.8 ± 1.3	—
Gestational age (weeks)	37.9 ± 1.7 (*n* = 41)	37.3 ± 3.0 (*n* = 54)	0.288	37.5 ± 2.5 (*n* = 95)	37.5 ± 2.8
Birth length *z* score	−4.1 ± 1.6 (*n* = 26)	−2.5 ± 1.4 (*n* = 38)	**<0.001**	−2.7 ± 1.0 (*n* = 64)	−4.1 ± 1.5
Birth weight *z* score	−3.0 ± 0.9 (*n* = 40)	−2.4 ± 1.0 (*n* = 54)	**0.009**	−3.1 ± 1.7 (*n* = 94)	−3.1 ± 1.2
Birth OFC *z* score	−1.2 ± 2.6 (*n* = 22)	−1.8 ± 1.7 (*n* = 30)	0.309	−1.5 ± 2.1 (*n* = 52)	−1.5 ± 1.1
Paternal age at childbirth (years)	33.7 ± 4.8 (*n* = 29)	34.4 ± 5.2 (*n* = 40)	0.590	34.1 ± 5.0 (*n* = 69)	—
Maternal age at childbirth (years)	31.3 ± 4.8 (*n* = 29)	31.7 ± 5.1 (*n* = 40)	0.755	31.6 ± 4.9 (*n* = 69)	—
Parameters at birth					
Weight ≤ 10th centile	98%	86%	**0.043**	91%	—
Length ≤ 10th centile	98%	84%	**0.026**	90%	—
Relative macrocephaly	86%	33%	**<0.001**	56%	84.6%
Postnatal course					
No catch-up growth; height ≤ 3rd centile	93%	88%	0.387	90%	92.3%
Normal head circumference; OFC ≥ 3rd centile and ≤97th centile	88%	60%	**0.001**	72%	—
Normal cognitive development	86%	68%	**0.041**	76%	76.9%
Asymmetry					
Face/body/limbs	77%	56%	**0.033**	65%	64.1%
Facial features					
Triangular shaped face	84%	88%	0.573	86%	—
High/bossing forehead	84%	79%	0.552	81%	92.3%
Other: e.g., small chin, thin lips, downturned corners of the mouth, late closure of fontanelle	67%	65%	0.794	66%	—
Other features					
Clinodactyly of the fifth finger	63%	63%	0.970	63%	64.1%
Genital abnormalities (e.g., cryptorchidism, hypospadias)	16%	14%	0.759	15%	—
Other: e.g., brachymesophalangy, syndactyly toes, inguinal hernia, pigmentary changes	33%	40%	0.429	37%	17.9%
Total score (maximum = 15)	11.3 ± 2.1	9.4 ± 1.2	**<0.001**	10.2 ± 1.9	—

SRS, Silver–Russell syndrome; OFC, occipitofrontal circumference. The *p* value < 0.05 is presented in boldface.

**Table 4:** Clinical features of 37 SRS subjects with IC1 hypomethylation and 10 SRS subjects with mUPD7.

Clinical Features	IC1 Hypomethylation (*n* = 37)	mUPD7 (*n* = 10)	*p* Value
Male/Female	23/14	4/6	0.217
Age at diagnosis (years)	4.4 ± 4.1	6.1 ± 7.4	0.355
Height *z* score at diagnosis	−3.0 ± 1.7	−3.8 ± 1.2	0.199
Weight *z* score at diagnosis	−3.0 ± 1.5	−3.2 ± 1.4	0.639
Gestational age (weeks)	38.0 ± 1.7 (*n* = 35)	36.6 ± 1.6 (*n* = 9)	**0.026**
Birth length *z* score	−4.1 ± 1.8 (*n* = 23)	−3.7 ± 0.9 (*n* = 5)	0.578
Birth weight *z* score	−3.0 ± 0.9 (*n* = 34)	−2.5 ± 0.9 (*n* = 9)	0.150
Birth OFC *z* score	−1.3 ± 1.3 (*n* = 20)	−1.6 ± 1.1 (*n* = 4)	0.800
Paternal age at childbirth (years)	32.9 ± 4.3 (*n* = 25)	37.6 ± 4.8 (*n* = 7)	**0.018**
Maternal age at childbirth (years)	30.3 ± 4.1 (*n* = 25)	35.3 ± 5.1 (*n* = 7)	**0.011**
Parameters at birth			
Weight ≤ 10th centile	97%	100%	0.609
Length ≤ 10th centile	100%	90%	0.053
Relative macrocephaly	89%	50%	**0.004**
Postnatal course			
No catch-up growth; height ≤ 3rd centile	92%	100%	0.363
Normal head circumference; OFC ≥ 3rd centile and ≤ 97th centile	89%	80%	0.451
Normal cognitive development	86%	80%	0.618
Asymmetry			
Face/body/limbs	76%	50%	0.120
Facial features			
Triangular shaped face	89%	50%	**0.004**
High/bossing forehead	81%	80%	0.940
Other: e.g., small chin, thin lips, downturned corners of the mouth, late closure of fontanelle	68%	60%	0.662
Other features			
Clinodactyly of the fifth finger	68%	20%	**0.006**
Genital abnormalities (e.g., cryptorchidism, hypospadias)	19%	0%	0.142
Others: e.g., brachymesophalangy, syndactyly toes, inguinal hernia, pigmentary changes	35%	10%	0.128
Total score (maximum = 15)	11.4 ± 2.2	8.7 ± 2.5	**0.001**

SRS, Silver–Russell syndrome; IC, imprinting center; mUPD7, maternal uniparental disomy of chromosome 7; OFC, occipitofrontal circumference. The *p* value < 0.05 is presented in boldface.

## Data Availability

Not applicable.

## Abbreviations

SRS: Silver–Russell syndrome, IC: imprinting center, mUPD7: maternal uniparental disomy of chromosome 7, ART: assisted reproductive technology, MS-MLPA: methylation-specific multiplex ligation-dependent probe amplification, MS-HRM: methylation-specific high-resolution melting.
